# Dental and oral manifestations of celiac disease

**DOI:** 10.4317/medoral.22506

**Published:** 2018-11-21

**Authors:** Izabela-Taiatella-Siqueira-Alves Cruz, Fabian-Calixto Fraiz, Adriane Celli, José-Miguel Amenabar, Luciana-Reichert-da-Silva Assunção

**Affiliations:** 1 Master in Dentistry, Postgraduate Student, Universidade Federal do Paraná, Curitiba, Brazil; 2 Doctor in Dental Science, Full Professor, Department of Stomatology, Universidade Federal do Paraná, Curitiba, Brazil; 3 Doctor in Child and Adolescent Health, Adjunct Professor, Department of Pediatrics, Universidade Federal do Paraná, Curitiba, Brazil; 4 Doctor in Dentistry, Associate Professor, Department of Stomatology, Universidade Federal do Paraná, Curitiba, Brazil; 5 Doctor in Pediatric Dentistry, Associate Professor, Department of Stomatology, Universidade Federal do Paraná, Curitiba, Brazil

## Abstract

**Background:**

The objective of this study was to evaluate the dental and oral manifestations in patients with celiac disease.

**Material and Methods:**

The sample consisted of 40 patients with the disease and 40 without the disease matched by age in southern Brazil. The CD group included patients previously diagnosed by positive anti-endomysial (IgA) examination and confirmed by small intestine biopsy. The presence of dental enamel defects and dental caries was evaluated by a calibrated researcher according to AINE’s and WHO’s criteria, respectively. The history of recurrent aphthous ulcers and dry mouth was obtained through reporting. For the evaluation of the salivary flow, the saliva samples were obtained through the non-stimulated and stimulated saliva collection method.

**Results:**

There was a significant association between CD and dental enamel defects (OR=2.38, *P*=0.045) and dry mouth (OR=9.15, *P*=0.002). No difference was found for the report of recurrent aphthous ulcers and caries experience between the two groups. Patients with CD had normal pattern of unstimulated and stimulated saliva flow rates (0.67 ± 0.38 ml / min and 1.14 ± 0.47 ml / min, respectively). A higher occurrence of dental enamel defects was observed in patients with classic CD (*P*=0.054). Of the 1,962 permanent teeth, 59 presented dental enamel defects, 71.8% of which were in patients with CD (*P*=0.001), predominantly in molars (*P*=0.009).

**Conclusions:**

CD increased the likelihood of dental enamel defects and dry mouth sensation. The oral examination can be an important auxiliary tool for the identification of cases of the disease.

** Key words:**Celiac disease, oral manifestations, dental enamel hypoplasia.

## Introduction

Celiac disease (CD) is an autoimmune disorder affecting both the epithelium and the lamina propria of the small intestine in individuals who are genetically susceptible and intolerable to gluten. Gluten sensitivity causes villous atrophy, which resolves with a gluten-free diet (1). When it is not diagnosed early, CD may lead to significant impact on quality of life, specially related to the clinical symptoms such irritable bowel syndrome (2) and psychiatric disorders (3).

The clinical characteristics of CD varies considerably. The classic type often occurs early in life and is characterized by intestinal malabsorption, including chronic diarrhea, weight loss, abdominal distension, and developmental delay. The non-classic type is characterized by few or no gastrointestinal symptoms and by extraintestinal manifestations, such as dermatitis herpetiformis, iron-deficiency anemia, short stature, cryptogenic hepatitis, osteoporosis and ataxia. There is also a third type: asymptomatic CD (4).

In addition to systemic manifestations of the disease, some clinical disorders of the oral cavity, such as dental enamel defects (DED), recurrent aphthous ulcers (RAU), and salivary disorders, may occur during the course of CD (5). Only one Finnish study involving individuals aged 3 to 86 years described a sensation of dry mouth in 29 (22.6%) of 128 CD patients and in 7 (23%) of 30 individuals without CD, yielding a nonsignificant difference (6).

Although it has been suggested that the oral manifestations of CD can help identify individuals with this disorder, especially those with the asymptomatic type (7), the association between oral manifestations and CD is still controversial. The causes of DED in CD patients are still unknown. Enamel hypoplasia may result from hypocalcemia induced by CD (8), from genetic susceptibility (9), or from an autoimmune reaction in the enamel organ during odontogenesis (10).

Another oral manifestation associated with CD is RAU; however, its etiology remains unclear (11). The possible explanation for this correlation is iron, folic acid, and vitamin B12 deficiencies, present in 20% of CD patients (12). The sensation of dry mouth may be associated with decreased salivary flow (12) or with the presence of Sjögren’s syndrome, a condition associated with some CD patients (13).

With respect to the prevalence of dental caries, some conflicting results have been reported. One study has described a lower prevalence of this disease in CD patients under treatment, which can be explained by the more balanced diet and the smaller number of snacks between meals (13). Other study, however, has not found any difference in the prevalence of caries between CD and healthy individuals (14).

Celiac disease is one of the most frequent types of food intolerance with a worldwide average prevalence of approximately 1% (1). A study carried out in Brazil have shown that the prevalence of celiac disease is considerably higher than previously resumed (15). The emergence of serological tests of high accuracy and greater attention to atypical manifestations of the disease has increased the prevalence of CD. Prevalence is estimated at around 1: 100 in the general population (16). The first epidemiological survey in Brazil for investigation of CD was carried out with 2,045 healthy blood donors, predominantly men, in which CD prevalence was 1:681 (15). A high prevalence of CD (1:417) was also observed in the city of Curitiba, Southern of Brazil, where 2,086 healthy individuals and blood donors were evaluated (17). While the few epidemiological data available in Brazil indicate a remarkable prevalence of CD, only one Brazilian study, conducted in the southeast region, assessed oral disorders in CD patients (11). The aim of this study was to investigate the prevalence and oral manifestations of CD in southern Brazil comparatively to a control group. Curitiba is located in southern Brazil and is largely populated by European descendants. Therefore, the undertaking of this study is justified, given that the manifestation of CD and its oral signs and symptoms can present different epidemiological patterns in specific populations (18).

## Material and Methods

-Study Population

The study was approved by the Human Research Ethics Committee of the Federal University of Paraná (protocol n. 41861015.0.0000.0102).

The exclusion criteria were participants who presented with fluorosis, use of fixed orthodontics and DED associated with other systemic diseases such as congenital erythropoietic porphyria, hemolytic anemia and chronic renal failure.

Forty patients with CD were selected from the Pediatric Gastroenterology Clinic of the Teaching Hospital of the Universidade Federal do Paraná, Curitiba, Brazil. The CD group included patients with a positive IgA anti-endomysial antibody test and diagnosis confirmed by small intestine biopsy associated with positive serology for CD.

A total of 100 patients treated at the pediatric dental practice affiliated with the Federal University of Paraná, Curitiba, Brazil was evaluated to compose the control group. Twenty-three patients with gastrointestinal symptoms and two with fluorosis were excluded, resulting in a sample of 75 patients. These patients were selected according to age resulting a total of 40 patients.

-Questionnaire

Medical, family and dental histories were collected using a previously tested questionnaire. The medical history investigated prenatal, perinatal, and postnatal events. The questions about the prenatal period included intercurrent events during pregnancy, such as use of medications, high-grade maternal fever and episodes of infection. Perinatal intercurrent events included problems at delivery, such as anoxia, cyanosis, and premature birth. Comorbidities that might have occurred in childhood, such as mumps, chickenpox, nutritional deficiencies, otitis, urinary tract infections, and bouts of high-grade fever, were also investigated. The use of antibiotics during childhood was also assessed.

The type of disease and age at diagnosis were evaluated. The classification of the type of CD was made based on the signs and symptoms reported by the patients or legal guardians and on medical records.

Dental history included questions about recurrent aphthae and their frequency, dry mouth and history of dental trauma.

-Pretest and calibration

In a pretest stage, the questionnaire was applied to 12 participants or their legal guardians whose ages were similar to those included in the present study for cultural adequacy and check of its applicability in the collection of data that allow meeting the objectives of the study. These individuals were not included in the final sample. Some words were replaced in order to improve the understanding of some questions.

One of the researchers (ITSAC) was previously calibrated for the clinical parameters used in the study. For DED calibration the AINE’s (1986) (19) classification was selected ( Table 1). Interrater and intrarater reliability yielded values greater than 0.826.

**Table 1:**
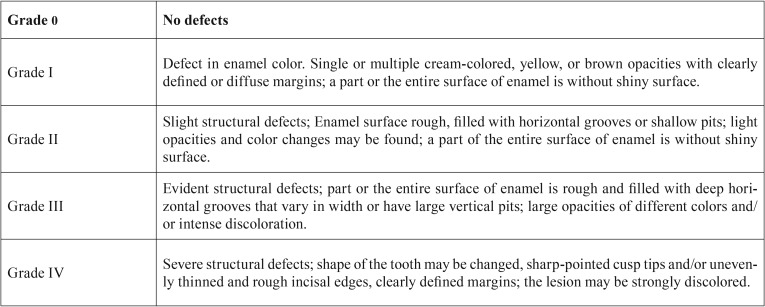
Classification of DED according to AINE (1986).

The method used for calibration of dental caries followed the criteria recommended by WHO (1997) (20), using DMFT (decayed and filled permanent teeth with indication for extraction) and dmft (decayed and filled deciduous teeth with indication for extraction). intrareader and interrater values were greater than 0.895.

-Clinical examination

The clinical examination was performed in a conventional chair under natural lighting using a flat surface mirror and a blunt-tipped probe after cleaning of dental surfaces with sterile gauze pads. Opacities were distinguished from white spot lesions based on color, texture, demarcation, and relationship to the gingival margin. DED that affect deciduous and permanent teeth were categorized according to Aine’s (1986) classification (19). Also, according to this classification, specific DED were defined as those which occurred chronologically in the four dental quadrants, whereas nonspecific DED did not occur chronologically.

Dental caries was also evaluated under the same environmental conditions used for DED. The frequency of untreated caries lesions (component “d” of dmft index, for deciduous teeth and “D” of DMFT index, for permanent teeth) was taken into consideration in the data analysis.

-Subjective xerostomia and salivary flow rate

Subjective xerostomia was evaluated through the question “does your mouth usually feel dry?” (21). The flow rates of unstimulated and stimulated whole saliva were determined using spit method. The collection of unstimulated saliva started with the instruction to accumulate the saliva in the floor of the mouth, without stimulation of saliva secretion by means of orofacial movements. The participants spat out into 10-mL plastic containers that were graded in 0.1-mL units for 5 minutes. After 5 minutes, participants were told to expectorate residual saliva into the container and unstimulated saliva flow rates were read. Stimulated saliva was collected by chewing on a piece of Parafilm (0.3 g of Parafilm M laboratory film, American National Can, Greenwich, CT). At 30-second intervals saliva was expectorated into graded containers, and after 5 minutes stimulated saliva flow rates were read. Total unstimulated and stimulated saliva flows were expressed in mL per minute. Unstimulated flow rates less than 0.1 mL/min were classified as unstimulated hyposalivation, while stimulated flow rates less than 0.7 mL/min were regarded as stimulated hyposalivation (22).

-Statistical analysis

The statistical analysis was made using Statistical Package for the Social Sciences (SPSS), version 19.0 (IBM SPSS Inc., Chicago, IL, USA). The variables were analyzed descriptively, including their absolute values and proportions. Wilcoxon’s test was used in the inferential analysis for the association between numerical and dichotomous categorical variables. Pearson’s chi-square test, or Fisher’s exact test, was used for assessment of two categorical variables. Odds ratio, with its respective 95% confidence interval, was also included in the analysis. The chi-square goodness-of-fit test was also used. The significance level was set at 5%.

## Results

Most participants were female (71.3%) with median age of 16.50 years. Mean age at diagnosis was 11.21 years (SD=8.84). For 82.1% of the patients, the diagnosis of CD was confirmed by biopsy before the age of 20 years.

Of 80 participants, 63 (78.8%) had permanent dentition, 14 (17.5%) had mixed dentition, and only 3 (3.8%) deciduous dentition. There was no statistically significant difference between DED and type of dentition (*P*=0.182). The presence of DED was not associated with age at CD diagnosis (*P*=0.102) and CD patients were 2.83 times more likely to have DED (*P*=0.045) ( Table 2). CD patients have normal unstimulated and stimulated salivary flow rate mean values (0.67±0.38 ml/min and 1.14±0.47 ml/min). Nevertheless, they have 9.15 times more likely to report dry mouth (*P*=0.002) compared to the control group. No statistically significant difference was observed between RAU and untreated caries experience ( Table 2).

**Table 2:**
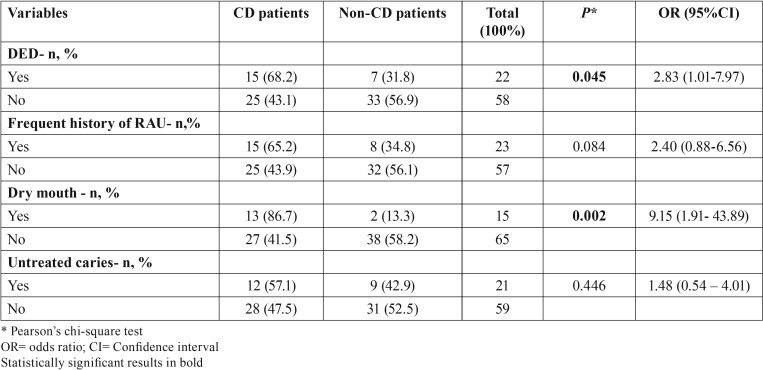
Oral manifestations in CD patients and control.

**Figure 1:**
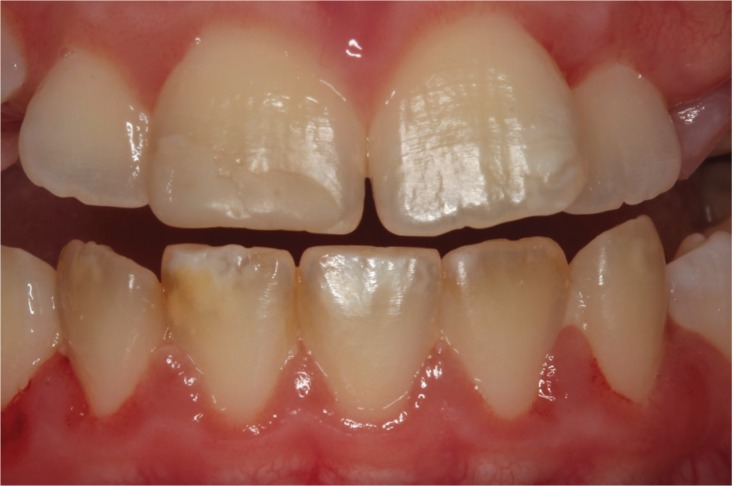
Grade I (Aine classification) dental enamel defects in an 8-year-old girl with celiac disease.

**Figure 2:**
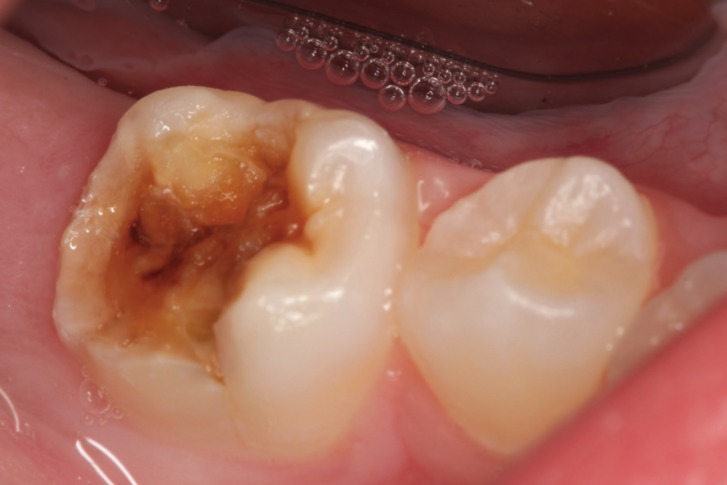
Permanent mandibular first molar with Grade IV (Aine classification) dental enamel defect.

Most specific and nonspecific defects were observed in CD patients (85.7% and 56.3%, respectively). Only specific defects were statistically significant (*P*=0.048), and CD patients had a higher prevalence of these defects than control individuals. Grade I defects (Fig. 1) were the most frequent in both groups, affecting a total of 20 individuals – 65% among CD patients and 35% among control individuals. Two patients with CD presented grade II defects and only one patient with the disease had grade IV defect (Fig. 2) ( Table 3).

**Table 3:**
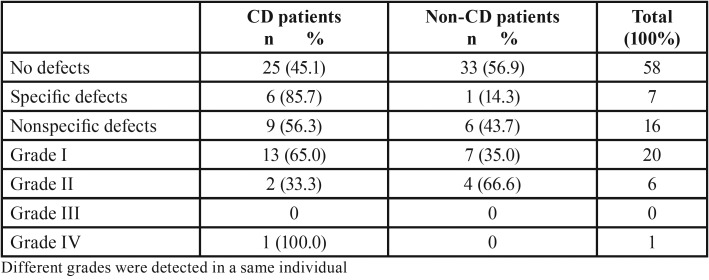
Distribution of different DED grades among CD patients and control.

Among CD patients, 15 (37.5%) presented with the classic form of the disease, 22 (55%) experienced nonclassic symptoms, and 3 (7.5%) developed the asymptomatic type. DED was detected in 7 (46.6%) patients with classic CD and in 8 (33.3%) individuals with the nonclassic or asymptomatic type. Only one patient with asymptomatic CD had DED.  Table 4 shows DED as a function of CD type. Those with the classic form of the disease tended to develop DED when compared with the non-CD group (*P*=0.054).

**Table 4:**
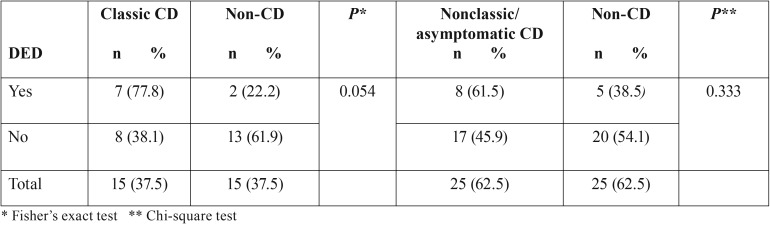
Distribution of DED cases as a function of CD type.

Taking into account only those individuals with DED, no significant differences in intercurrent events in the prenatal, perinatal, and postnatal periods were noted between the groups (*P*>0.05). Likewise, history of dental trauma was not associated with DED in any of the groups (*P*=1.000).

A total of 1,962 permanent teeth (984 from CD patients and 978 from control group) and 163 deciduous teeth (79 from CD patients and 84 from control group) were evaluated. Of the permanent teeth 59 had DED, of these 42 (71.2%) were from CD patients and 17 (28.8%) were from control group (*P*=0.001). Among the permanent teeth, molars were the teeth most frequently affected by DED followed by incisors and canines. The prevalence of DED in molars was higher among CD patients than in control group (n=22 and n=7, respectively, *P*=0.009). Also in incisors, the prevalence of DED was higher among CD patients than in control group (n=17 and n=4, respectively, *P*=0.008). A total of 6 canines was affected (n=2 in CD patients and n=4 in the control group) and only 3 premolars presented DED (n=2 in CD patients and n=1 in the control group), however with no statistically significance between the groups.

Out of 163 deciduous teeth, only three (1.84%) had DED, being two deciduous incisors from CD patients and 1 second deciduous molar from control group.

## Discussion

This study assessed dental and oral manifestations of CD. Results showed that CD patients were 2.83 times more likely to have DED than control. Studies from other countries that used the same classification and diagnostic criteria also demonstrated this relationship (14,19). This indicates the importance of oral assessment as a tool for the diagnosis of CD even in patients with no signs and symptoms, since the disease may be asymptomatic (23).

Among DED types, there was a higher prevalence of specific defects in CD patients than in control. These findings are consistent with other study which also detected a higher prevalence of this type of defect in CD patients (11).

Grade I DED was the most prevalent type among CD patients when compared to the control group. Other authors also found a higher prevalence of these defects in CD patients (11,14). Only one study described a higher prevalence of grade II DED (24), which is in according to our findings, pointing out that grade II defects were more prevalent in patients without CD. On the other hand, the prevalence of the most severe type of defect (Grade IV) was more rare and observed only in one CD patient, corroborating the findings of a previous study (24).

DED tended to affect patients with the classic form of CD more frequently. We believe this difference could have been further investigated if the sample size were larger. Few studies have looked into this association. A previous study observed DED in 30.9% of individuals with the classic form of CD and in 100% of individuals with nonclassic or asymptomatic symptoms (4). In the present study, only 33.3% of the individuals with nonclassic or asymptomatic CD had DED, and among those, only one patient with the asymptomatic type had DED. This important finding should be investigated further, especially by studies targeted at the screening of CD.

The number of teeth with DED was significantly higher in CD patients, and this may be explained by the higher prevalence of specific defects, i.e., defects that affect the four dental quadrants (12,11). These findings are in according with other studies (11,14).

Involvement of permanent molars and incisors due to DED was noted among CD patients when compared to the control group. Other study showed the same frequency of teeth affected by DED (25). The North American Society for Pediatric Gastroenterology, Hepatology, and Nutrition acknowledges the presence of DED in permanent dentition as a sign of CD (26). The literature describes an association between age at CD diagnosis and the involvement of specific groups of permanent teeth (11). However, this could not be confirmed by the present study as the mean age at CD diagnosis was 11.2 years, which could possibly be related to an association of DED with other groups of teeth. Nevertheless, another possible explanation for the larger involvement of incisors and molars in this study could be the likelihood of DED among patients with the classic form of CD, which is characterized by diagnosis at younger ages. Further studies are needed to elucidate this association.

Children with CD had only 2.53% of DED in deciduous teeth. This low prevalence of defects can be explained by the mineralization of the crown in deciduous teeth, which begins around the fourth month of intrauterine life and ends at approximately the tenth month after birth (4), when most individuals still have a gluten-free diet.

The etiology of DED in gluten-intolerant patients remains unknown, but some authors link these defects to the malabsorption syndrome, caused by tiny lesions in the small intestine and eventually lead to deficiency of other nutrients that are essential to the development of the tooth germ (26). It is also suggested that problems with amelogenesis in the teeth of CD patients may be caused by autoimmune mechanisms, such as the action of human leukocyte antigens (HLA-DQ-8 and HLA-DR3) upon enamel organs (10).

There was no association between DED and comorbidities in the prenatal, perinatal, and postnatal periods. There is only one study that investigated this association (24). Such study was conducted with Dutch children with a mean age of 9.7 years in the group of CD patients and of 10 years in the control group. In that study, gestational diabetes, premature birth, long bouts of high-grade fever, use of antibiotics, and dental trauma were not associated with DED. The lack of association between intercurrent events in the three assessed periods may be due to the recall bias during the questionnaire application, especially among older individuals. However, the pretest allowed adjusting the questions, minimizing any negative effects.

The prevalence of RAU in the general population is estimated at 5 to 60%; and 20% of the general population will be affected by this disorder sometime during their lifetime (10). However, previous studies suggest a higher prevalence of RAU in CD patients (11,25). The association between RAU and CD has been claimed to stem from specific autoimmune conditions (27). With the interruption of a gluten-rich diet, there is significant improvement or even total remission (10). Nevertheless, in the present study, there was no association between RAU and CD, which is in line with the findings of a previous study (28).

CD patients were 9.15 times more likely to present with dry mouth than control. One study observed that CD patients often complain of a sensation of dry mouth (6), but there was no statistical significance. In a study with Brazilian patients, salivary flow decreased by 36% among CD patients (11). However, a Turkish study has found similar results in salivary flow among CD and healthy patients (29). With respect to the current findings, it can suggest that dry mouth sensation may be present even when salivary flow is normal. Xerostomia might be not only related to the salivary flow rate, but also to the organic and inorganic contents of the saliva (30). Therefore, further studies are suggested to evalua-te quality parameters presented in the saliva of CD patients.

The association between caries experience and CD is still controversial. In a study with Italian CD patients, observed an increase in the prevalence of dental caries (25). According to those authors, dental fragility caused by hypoplasia and changes in salivary flow may increase the risk of caries lesions in CD patients. On the other hand, a previous study described lower caries experience among CD patients, attributing this finding to a stricter diet, which restricts the consumption of gluten, found in several cariogenic foods (11). Notwithstanding, a nonsignificant association between dental caries and CD has been reported in the literature (14), corroborating the findings of this study.

## Conclusions

It can be concluded that DC was associated with the occurrence of DED and dry mouth sensation. Oral clinical examination becomes an important tool in the diagnosis and screening of cases of patients with celiac disease.
